# Real-Time Monitoring of Blood Pressure Using Digitalized Pulse Arrival Time Calculation Technology for Prompt Detection of Sudden Hypertensive Episodes During Laryngeal Microsurgery: Retrospective Observational Study

**DOI:** 10.2196/13156

**Published:** 2020-05-15

**Authors:** Yong-Seok Park, Sung-Hoon Kim, Yoon Se Lee, Seung-Ho Choi, Seung-Woo Ku, Gyu-Sam Hwang

**Affiliations:** 1 Department of Anesthesiology and Pain Medicine Asan Medical Center University of Ulsan College of Medicine Seoul Republic of Korea; 2 Department of Otolaryngology Asan Medical Center University of Ulsan College of Medicine Seoul Republic of Korea

**Keywords:** larynx, blood pressure, photoplethysmography, pulse

## Abstract

**Background:**

Laryngeal microsurgery (LMS) is often accompanied by a sudden increase in blood pressure (BP) during surgery because of stimulation around the larynx. This sudden change in the hemodynamic status is not immediately reflected in a casual cuff-type measurement that takes intermittent readings every 3 to 5 min.

**Objective:**

This study aimed to investigate the potential of pulse arrival time (PAT) as a marker for a BP surge, which usually occurs in patients undergoing LMS.

**Methods:**

Intermittent measurements of BP and electrocardiogram (ECG) and photoplethysmogram (PPG) signals were recorded during LMS. PAT was defined as the interval between the R-peak on the ECG and the maximum slope on the PPG. Mean PAT values before and after BP increase were compared. PPG-related parameters and the correlations between changes in these variables were calculated.

**Results:**

BP surged because of laryngoscopic manipulation (mean systolic BP [SBP] from 115.3, SD 21.4 mmHg, to 159.9, SD 25.2 mmHg; *P*<.001), whereas PAT decreased significantly (from mean 460.6, SD 51.9 ms, to 405.8, SD 50.1 ms; *P*<.001) in most of the cases. The change in SBP showed a significant correlation with the inverse of the PAT (*r*=0.582; *P*<.001). Receiver-operating characteristic curve analysis indicated that an increase of 11.5% in the inverse of the PAT could detect a 40% increase in SBP, and the area under the curve was 0.814.

**Conclusions:**

During LMS, where invasive arterial catheterization is not always possible, PAT shows good correlation with SBP and may, therefore, have the potential to identify abrupt BP surges during laryngoscopic manipulations in a noninvasive manner.

## Introduction

### Background

Laryngeal microsurgery (LMS) is often accompanied by a sudden increase in blood pressure (BP) during surgery because of stimulation around the larynx [1,2]. Laryngoscopic manipulations cause sympathetic nervous system stimulation and increase in the level of plasma catecholamines, such as epinephrine and norepinephrine [3]. Acute hypertension resulting from this process can cause complications such as ischemic heart disease, heart failure, stroke, and life-threatening arterial bleeding even in patients without predisposing factors [4,5]. Moreover, myocardial ischemia or arrhythmia can occur during the perioperative period because of the stimulation of the deep receptors of both the larynx and cardioinhibitory fibers of the vagus nerve [6]. There have been previous studies regarding the appropriate methods of anesthesia for maintaining a stable hemodynamic status in patients during LMS [7,8]. Despite a sudden change in the hemodynamic status, an abrupt BP rise is not immediately reflected in a casual cuff-type measurement that takes intermittent readings every 3 to 5 min.

Pulse arrival time (PAT), measured as the interval from the R-peak on an electrocardiogram (ECG) to the peripheral arrival of the pulse wave, has been used as an estimate for pulse transit time and may be a noninvasive marker for BP [9-14]. Recent advancements in technology enabled adjustment for confounding factors (eg, heart rate, arterial stiffness, and pre-ejection time) in estimating BP from PAT [9,15,16]. Furthermore, a cuff-less approach for 24-hour BP monitoring became possible using wearable devices [13]. Clinical application and commercialization of these wearable BP-monitoring devices are underway [17,18].

### Objectives

We previously reported that beat-to-beat changes in PAT can effectively detect decreases in systolic BP (SBP) during anesthesia induction in hypertensive patients undergoing renal transplantation [11]. However, most previous studies have estimated the state of hypotensive events, but no report to date has focused on PAT monitoring to detect an abrupt BP surge. In this study, we investigated whether noninvasive PAT could be used to monitor rapid BP increases during LMS in real time.

## Methods

### Study Population and Anesthesia Protocol

We retrospectively analyzed the electronic records of 30 patients with vocal cord polyps or edema who were scheduled for an elective LMS under general anesthesia. Patients with cardiac arrhythmias or incomplete data were excluded.

All patients were prepared for general anesthesia according to our institutional protocol. Premedication was not given. Cardiovascular medication, including antihypertensive medication, was administered until the day of surgery, except for angiotensin II receptor blocker or angiotensin-converting enzyme inhibitor. Routine monitoring included noninvasive intermittent BP (NIBP), ECG, pulse oximetry, and end-tidal concentration of CO_2_ using a multiparameter monitor (Philips IntelliVue MP70; Philips). All data were recorded simultaneously throughout the procedure. Volatile induction and maintenance of anesthesia were performed. Anesthesia was induced with 6% to 8% sevoflurane and rocuronium (0.5-1 mg/kg) and maintained with 2% sevoflurane and 50% nitrous oxide in oxygen.

### Data Acquisition and Signal Processing

ECG and photoplethysmogram (PPG) waveform data were recorded with data acquisition software (Vital Recorder) [19] at a sampling rate of 300 Hz, and NIBP was measured and recorded every 3 min. The signal data were transferred via serial port from the MP70 monitor to a computer running the Vital Recorder and written to the hard drive. PAT was defined as the interval between the R-peak on the ECG and the point at which the maximal rising slope appears on the PPG [11]. The PAT of each beat was calculated using the filter function, which was built using Python code in the Vital Recorder software (Multimedia Appendix 1). When the filter function is executed, the PAT at every heartbeat is calculated from the recorded ECG and PPG waveforms by the algorithm, and the PAT values at each time point are added as a new time series variable. The PPG waveform data recorded with the data acquisition software were converted to European Data Format [20] and analyzed with signal processing software (LabChart 8; AD Instruments) to acquire features of the PPG waveform, such as height, width_50_ (width at 50% height of each PPG wave peak), maximum slope, minimum slope, and area of each wave peaks [21]. These features were extracted by the peak analysis function of the software. The 1-min averages of each value just before and after laryngoscopic manipulation were calculated by the function and recorded for further analysis. All the acquired parameters during the laryngoscopic manipulation were compared. The 1-min averages of PAT just before laryngoscope insertion and at the lowest point of PAT after stimulation were used as the PAT parameters for each time point. PPG and PAT parameters were obtained simultaneously.

### Statistical Analysis

All study data are presented as mean (SD), n (%), or median (IQR). A Shapiro-Wilk test was used as a test of normality. A paired *t* test or Wilcoxon signed-rank test was used to compare parameters before and after LMS. The correlation between the changes in BP and PAT and between the changes in BP and PPG-related variables at all time points were evaluated with a Pearson correlation coefficient or Spearman correlation coefficient, as appropriate. Receiver operating characteristic (ROC) curve analyses were performed to evaluate the ability of PAT and PPG-related variables to detect a 40% change in the SBP. R version 3.4.2 software (R Foundation for Statistical Computing) was used for all statistical analyses.

### Ethics Approval and Consent to Participate

This study was approved by the institutional review board at Asan Medical Center (IRB No, 2017-2268). The requirement for written informed consent was waived because of the retrospective nature of the study and the anonymity of the biosignals used in this study.

### Availability of Data and Materials

All data generated or analyzed during this study are included in this published paper and Multimedia Appendices 1 and 2.

## Results

### Changes in Hemodynamic Variables and Photoplethysmogram Variables

**Figure 1:**
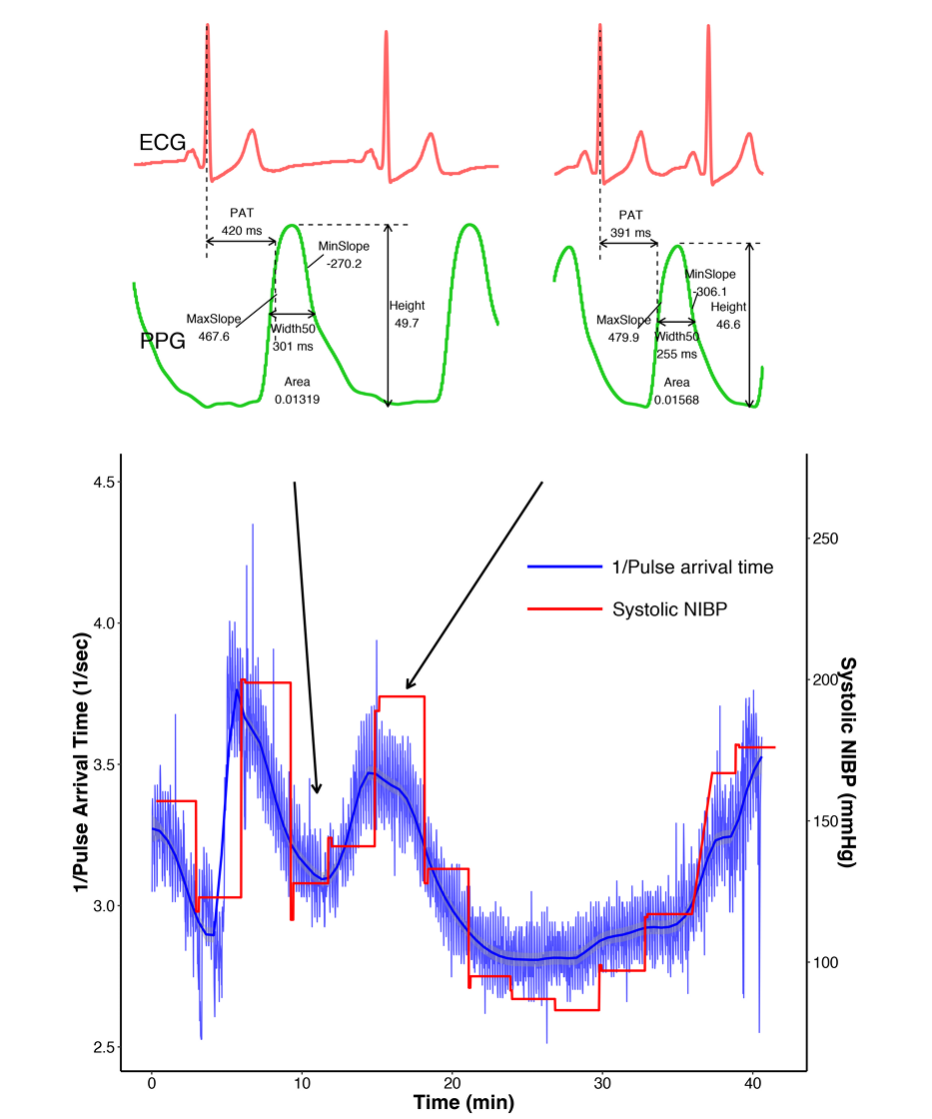
Tracing showing the relationship between systolic noninvasive intermittent blood pressure and pulse arrival time during laryngeal microscopic surgery. Beat-to-beat changes in pulse arrival time instantaneously reflected marked perturbations of systolic noninvasive intermittent blood pressure during endotracheal intubation and the beginning of the laryngeal microsurgery procedure. The insets show the electrocardiography (upper) and the photoplethysmographic waveform (bottom) results before and after the insertion of the laryngeal microscope. The thick blue line is the smoothed line of the PAT using locally estimated scatterplot smoothing.

**Figure 2:**
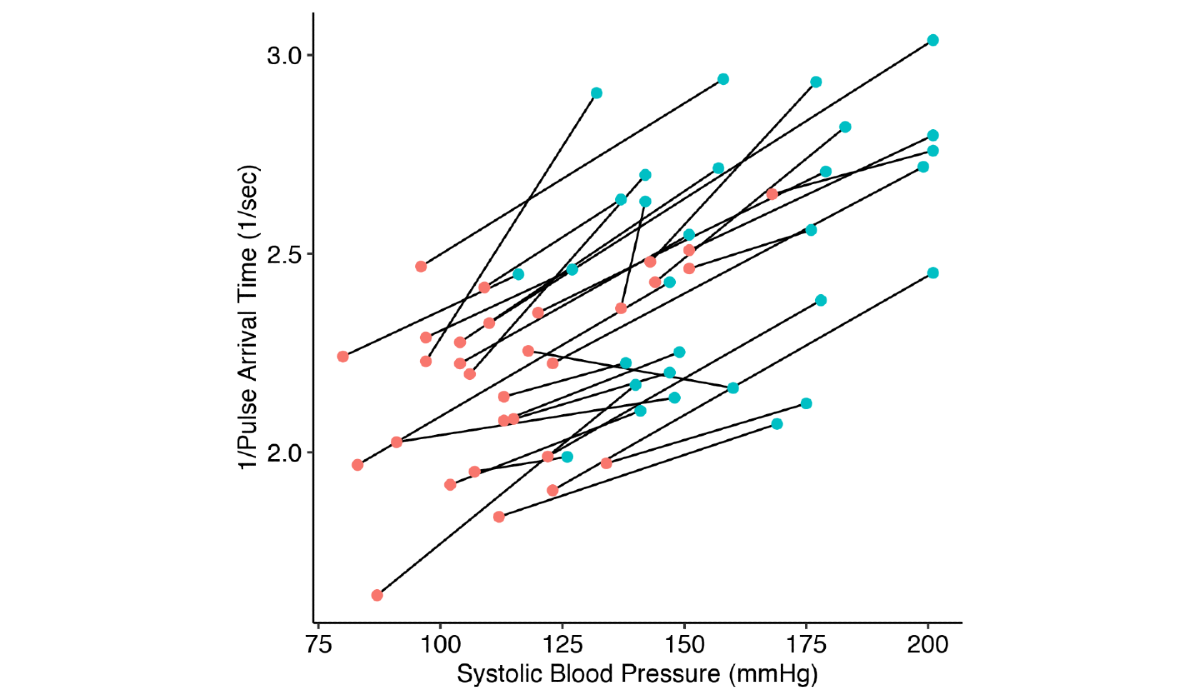
Changes in systolic blood pressure and pulse arrival time before and after surgical stimulation because of laryngeal microsurgery. Red dots represent changes before stimulation, whereas blue dots represent changes after stimulation.

After data processing, 60 sets of BP, ECG, and PPG values were analyzed. The demographic data of study patients are presented in Table 1. A representative plot of SBP and PAT of a randomly selected patient is shown in Figure 1. As indicated in Figure 1, systolic NIBP showed a tendency to follow the changing trends in the inverse of the PAT at intervals of 1 to 2 min, as the cuff-type NIBP device measures arterial pressure intermittently. Laryngoscopic manipulation during LMS caused a mean 41.3% increase in SBP and a mean 13.4% decrease in PAT. The inverse of the PAT increased as SBP increased in all patients except one (Figure 2). Heart rate increased by an average of 31.3% from baseline, and morphological parameters of PPG (eg, maximum slope, minimum slope, width, and area) also changed significantly after laryngoscope insertion (Table 2).

**Table 1:** Demographic and clinical characteristics of the 30 study patients.

Patient characteristics and prior comorbidities	Values
Sex, male, n (%)	18 (60)
Age (years), mean (SD)	58 (14)
Height (cm), mean (SD)	161.2 (7.6)
Weight (kg), mean (SD)	65.3 (12.2)
BMI (kg/m^2^), mean (SD)	24.2 (2.9)
Hypertension, n (%)	12 (40)
Diabetes mellitus, n (%)	4 (13)
Calcium channel blocker, n (%)	9 (30)
Beta blocker, n (%)	2 (6)
Angiotensin II receptor blocker, n (%)	3 (10)

**Table 2:** Changes in hemodynamic, photoplethysmographic, and pulse arrival time variables during blood pressure surge (N=30).

Variables	Before	After	*P* value
Systolic blood pressure (mm Hg), mean (SD)	115.3 (21.4)	159.9 (25.2)	<.001
Diastolic blood pressure (mm Hg), mean (SD)	69.0 (14.2)	97.6 (15.6)	<.001
Mean arterial blood pressure (mm Hg), mean (SD)	79.8 (14.7)	113.2 (17.0)	<.001
Heart rate (bpm), mean (SD)	78.1 (11.7)	101.3 (12.3)	<.001
Pulse arrival time (ms), mean (SD)	460.6 (51.9)	405.8 (50.1)	<.001
PPG^a^ amplitude (AU^b^), median (IQR)	45.6 (44.6-46.8)	46.3 (45.0-47.0)	.49
PPG maximum slope (AU/s), mean (SD)	485.0 (81.9)	564.3 (81.1)	<.001
PPG minimum slope (AU/s), mean (SD)	−247.2 (49.8)	−337.9 (50.3)	<.001
PPG width_50_ (ms), median (IQR)	256.8 (233.3-278.4)	221.9 (209.4-240.0)	<.001
PPG area (AU·ms), median (IQR)	15.7 (14.6-16.4)	17.0 (15.9-18.5)	.001

^a^PPG: photoplethysmogram.

^b^AU: arbitrary unit.

### Correlations between Hemodynamic Variables and Photoplethysmogram Variables

During the abrupt BP rise because of laryngeal stimulation, changes in the inverse of the PAT showed better correlation with the changes in SBP (*r*=0.582; 95% CI 0.281-0.779) than with changes in either mean arterial pressure (MAP; *r*=0.525; 95% CI 0.204-0.745) or diastolic BP (DBP; *r*=0.442; 95% CI 0.098-0.692). However, these differences in correlation coefficients were not statistically significant when compared with the coefficient between changes in the inverse of PAT and SBP using Fisher Z-transformation (correlation coefficient between changes in the inverse of PAT and MAP, *P*=.76; between changes in the inverse of PAT and DBP, *P*=.48).

**Figure 3:**
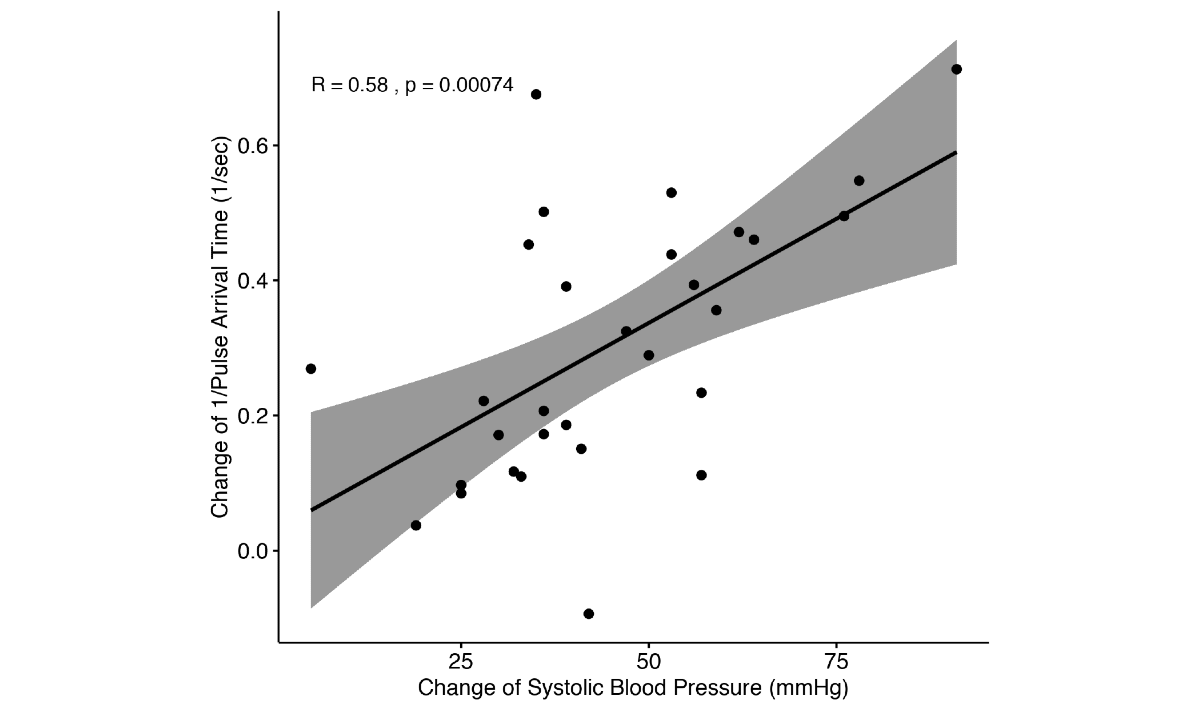
Correlation between the changes in systolic blood pressure and the changes in the inverse of the pulse arrival time (r=0.582, 95% CI 0.281-0.779; *P*<.001).

When we examined the relationship between SBP and PPG-related variables, the changes in the maximum slope of the PPG showed a significant correlation with SBP changes (*r*=0.49, *P*=.01). SBP changes also showed a good correlation with changes in the inverse of PAT (*r*=0.582; *P*<.001; Figure 3). Other PPG parameters such as amplitude, minimum slope, width_50_, and area did not show a statistically significant correlation with SBP changes (Table 3).

**Table 3:** Correlation between systolic blood pressure changes and photoplethysmogram-related variables.

Variables	Correlation coefficient, *r*	*P* value
Δ1/pulse arrival time	0.582	<.001
ΔPPG^a^ amplitude	0.338	.07
ΔPPG maximum slope	0.468	.009
ΔPPG minimum slope	−0.149	.43
ΔPPG width_50_	0.049	.79
ΔPPG area	0.254	.18

^a^PPG: photoplethysmogram.

**Figure 4:**
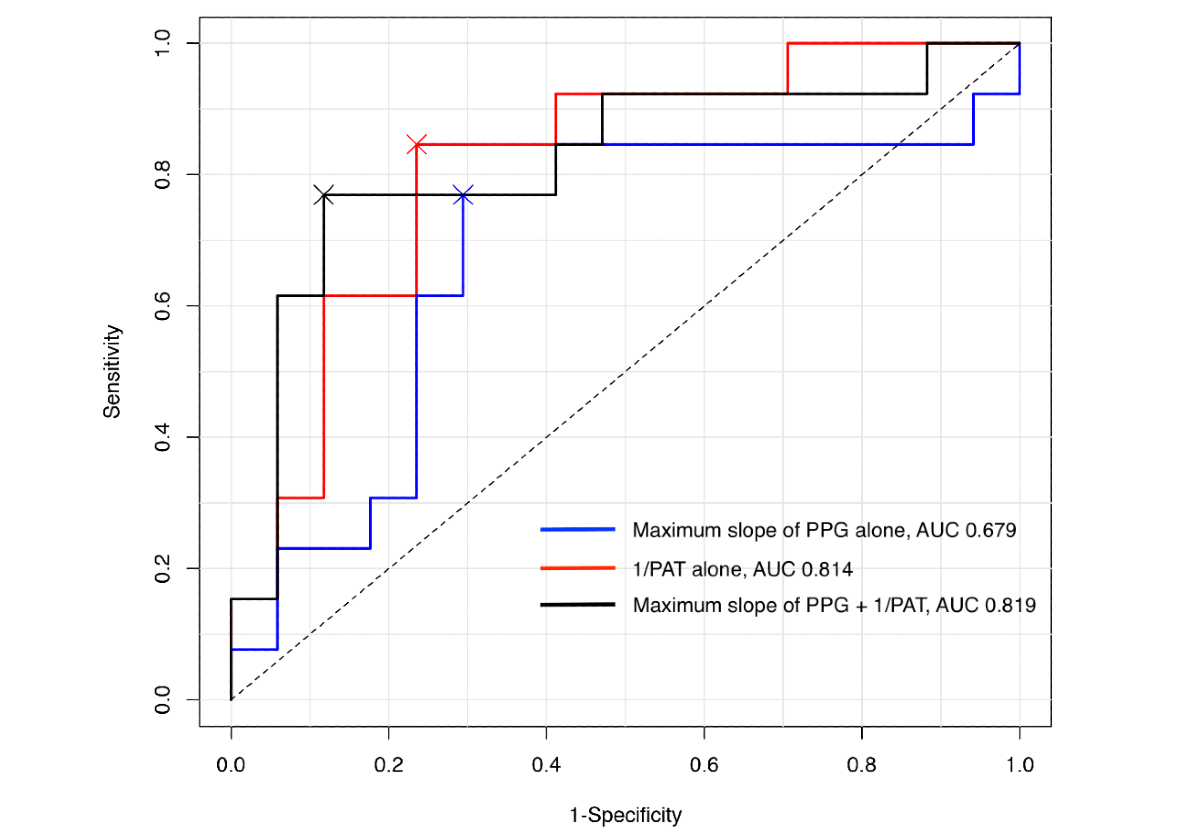
Receiver operating characteristic (ROC) curve analysis of percentage changes in the inverse of the pulse arrival time (PAT) and the maximum slope of photoplethysmogram (PPG) relative to 40% or greater changes in systolic blood pressure. Model using the maximum slope of PPG alone, area under the curve (AUC) 0.679, 95% CI 0.465-0.892, optimal cutoff value 11.7%; model using the inverse of the PAT alone, AUC 0.814, 95% CI 0.656-0.973, optimal cutoff value 11.5%; model using both inverse of the PAT and PPG maximum slope, AUC 0.819, 95% CI 0.650-0.988. Delong test for ROC curves of the model using inverse of PAT alone and the model using both inverse of PAT and PPG maximum slope, z=−0.114; *P*=.91.

PAT and PPG maximal slope showed a significant correlation with the changes in SBP, and in ROC curve analysis, these were evaluated as measurements for detecting greater than 40% change in SBP. In total, 3 models were introduced for the evaluation. The first model detected a 40% change in BP with 1/PAT alone. The second model used the maximum slope of PPG. The last model used both. The model that used changes in the 1/PAT alone to detect 40% or greater changes in the SBP showed an area under the curve (AUC) of 0.814 (95% CI 0.656-0.973 and optimal cutoff value 11.5%), whereas the model that used changes in the maximum slope of PPG showed an AUC of 0.679 (95% CI 0.465-0.892 and optimal cutoff value 11.7%). The combined ROC curve using the changes in both 1/PAT and PPG maximum slope showed an AUC of 0.819 (95% CI 0.650-0.988), which was not significantly different from the model with 1/PAT alone (Delong test, z=−0.114; *P*=.91; Figure 4).

## Discussion

### Principal Findings

We found that beat-to-beat PAT measurements using ECG and PPG are useful noninvasive indicators of BP surge in patients undergoing LMS. Specifically, changes in the inverse of the PAT and systolic NIBP show similar trends, and the inverse of the PAT changes shows a better correlation with the systolic NIBP changes than with those in the MAP or DBP. Moreover, beat-to-beat changes in the PAT reflect the BP surge 1 to 2 min earlier compared with cuff-type NIBP measurements.

Laryngoscopic manipulation in our study is clinically similar to the endotracheal intubation phase in our previous study [11]. PAT did not significantly decrease despite a 24.5% increase in SBP in the previous study. However, this study showed significant changes in SBP and PAT caused by laryngoscopic manipulation. The difference in the intensity of stimulation between LMS and endotracheal intubation might be an explanation for this discrepancy. In addition, the difference in the study population is a possible cause. All subjects of the previous study were hypertensive patients with end-stage renal disease, whereas only 40% of patients in this study had hypertension. It is assumed that the disturbances in autonomic cardiovascular regulation by chronic hypertension can cause these different responses to similar sympathetic stimulation [22].

Although changes in the maximum slope of the PPG showed a correlation with SBP changes, it did not increase the AUC of the ROC curve when it was combined with 1/PAT changes. Rather, the change in the 1/PAT value alone was sufficient to detect a 40% or greater change in the SBP, and the AUC of the ROC curve (AUC 0.814; Figure 4) was comparable with that in our previous study (AUC 0.85) [11]. This suggests that simply combining the features from the PPG signal and PAT does not further increase the diagnostic ability regarding detecting BP changes.

### Clinical Applicability of Pulse Arrival Time

LMS is a hemodynamically challenging procedure for anesthesiologists. Laryngoscopic manipulation and suspension of the larynx cause plasma catecholamine levels to rise and induce hemodynamic perturbation, including hypertension, tachycardia, bradycardia, and arrhythmias [7,23-27]. These hemodynamic aberrations can potentially produce poor postoperative outcomes, including prolonged hospital stay and morbidity [28]. As LMS has a short operating time and is usually an outpatient-based procedure, it is not suitable for invasive monitoring such as arterial catheterization unless the patient has known significant preoperative risk factors. However, it is not possible to screen every patient during routine preoperative evaluation for risks such as undiagnosed intracranial aneurysm. Even in patients without any underlying disease, unexpected changes in BP can be a potential risk for including bleeding, cerebrovascular events, or myocardial ischemia. Hence, simple and noninvasive methods of continuous monitoring are needed for early detection and quick response to unexpected BP change.

During different surgical procedures, various kinds of biosignals can be used to monitor abrupt physiological changes or to predict outcomes. PPG signals, in particular, can be used to reflect various physiological changes such as vascular compliance during hepatic graft reperfusion period following liver transplantation [29] and the success of lumbar sympathetic ganglion block [30]. There have been previous studies on the association between pulse transit time and BP. One study reported that the pulse transit time decreased as the DBP increased because of epinephrine injections in a cohort of 10 dogs [10]. In another study of 10 normal volunteers and 4 hemodialysis patients, PAT was found to correlate with SBP in normal subjects and could detect BP variations in dialysis patients [31]. Ambulatory BP monitoring based on PAT was reported to have significant accuracy in detecting BP above a threshold during nighttime [13]. In a previous evaluation of a monitoring device that estimates BP using a PAT derived by ECG and PPG, the accuracy of the instrument was comparable with that of a monitor based on oscillometry [32]. However, the relationship between BP and PAT is impaired in patients with chronic heart failure, whereas PAT is directly proportional to BP in healthy volunteers [33]. In addition to underlying heart disease, other possible confounding factors, including heart rate, pre-ejection period, and arterial stiffness, should be considered as limitations of BP monitoring using PAT [9,16,34,35]. In an earlier study of patients undergoing maxillofacial surgery, BP estimation using PAT was enhanced when two confounding factors, heart rate and arterial stiffness, were included in the multiple regression analysis model [9]. In this study, one subject showed a PAT increase from 443.4 to 462.6 ms despite a BP surge (systolic NIBP 118 to 160 mmHg; Figure 2). This was thought to be because of a decreased heart rate in this individual (102-75 bpm), which increased with an elevated BP in all other cases. This finding suggests that the refinement of the PAT-based BP-monitoring approach is necessary to reflect confounding factors.

**Figure 5:**
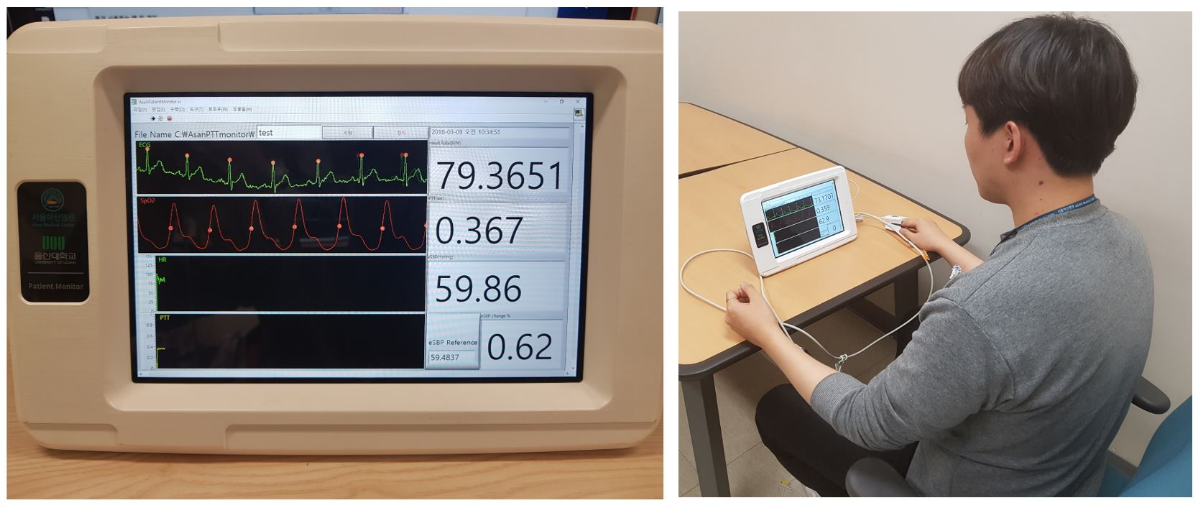
Development of a built-in device to monitor blood pressure surge using electrocardiogram (ECG) and photoplethysmographic (PPG) signal. Tablet personal computer as display and other biosignal sensor system were combined together and enclosed by housing. Blood pressure estimation algorithm was applied to the device, and the software was based on LabVIEW programming. Note that real-time–plotted red dots over ECG and PPG wave on display monitor indicate peak and first derivatives of those signals.

There were some notable findings from this analysis. First, we found that BP changes could be detected 1 to 2 min earlier with PAT than with NIBP, suggesting that PAT monitoring will facilitate earlier responses to BP changes than NIBP monitoring alone. Second, changes in PPG maximum slope also correlated with SBP changes. It is noteworthy that a feature of the PPG waveform alone correlates with BP, regardless of other signals, such as ECG. On the basis of the results of this study, we developed a built-in device, which is able to monitor BP surge using ECG and PPG signal in a continuous and noninvasive manner (Figure 5). Now we are preparing for prospective research to validate the accuracy and to evaluate the usefulness of this device in real clinical practice. We also expect to apply more advanced mathematical techniques to build up a plausible physiologic model of BP estimation. In a recent study that estimated BP from features of the PPG waveform using an artificial neural network, all estimations showed a good correlation with the reference values when the algorithm was validated [36]. Additional research on features of the PPG waveform itself in the estimation of BP is also likely to be of value.

### Limitations

Our study has several limitations of note. First, we used PAT measured by ECG R-wave and PPG signal to estimate actual pulse transit time, which should be measured by the time delay between arterial waveforms from two different places. Although the measurement of PAT is noninvasive and is technically easy to perform at low cost, it has been suggested that the PAT may not be a proper surrogate for the correct pulse transit time [37,38]. To calculate the exact pulse transit time, pre-ejection period should be subtracted from the PAT. However, the pre-ejection period was not measured in this study. Future studies are needed to measure the pre-ejection time, using a method such as the cardiac bioimpedance technique [35], and to investigate its hemodynamic relationships. Second, despite the importance of the heart rate as a confounding factor in the relationship between PAT and BP [9,15,34], adjustment for the heart rate was not performed in this study. The development of techniques to make adjustments for the effect of heart rate on PAT is likely to be meaningful. Third, we used an automated oscillometric NIBP as a routine BP monitor in our patients undergoing LMS. The oscillometric NIBP may not be reliable for measuring actual BP beyond a specific range [39]. However, with advances in technology and manufacturers’ efforts to meet medical standards, the accuracy of oscillometric NIBP instruments has been validated [40] and is considered to be reliable in determining BP measurements within the scope of our study. Fourth, instead of performing a calibration of PAT for NIBP, we only assessed the performance of PAT in monitoring relative changes in BP during the intubation period. PAT requires calibration to provide absolute B*P* values because both measurements have different scales and wide ranges of interpatient variability when they are converted to each other. Finally, as we conducted a retrospective analysis, selection bias or unknown confounders cannot be excluded. Well-designed prospective analyses and further device evaluation are warranted in the future.

### Conclusions

There is a clear correlation between BP and PAT, which demonstrates the potential of PAT as a useful and noninvasive means of continuous BP monitoring in situations where invasive monitoring is not appropriate. Further studies to adjust for confounding factors are needed to refine the BP-monitoring approach using PAT.

## Appendix

Multimedia Appendix 1 An add-on Python code for calculation of pulse arrival time which is included as a filter function in Vital Recorder software.

Multimedia Appendix 2 Raw data that were analyzed in this study.

## Abbreviations

AUC: area under the curve
BP: blood pressure
DBP: diastolic blood pressure
ECG: electrocardiogram
LMS: laryngeal microsurgery
MAP: mean arterial pressure
NIBP: noninvasive intermittent blood pressure
PAT: pulse arrival time
PPG: photoplethysmogram
ROC: receiver operating characteristic
SBP: systolic blood pressure
